# The role of astrocytes in prion-like mechanisms of neurodegeneration

**DOI:** 10.1093/brain/awab366

**Published:** 2022-03-10

**Authors:** Phillip Smethurst, Hannah Franklin, Benjamin E Clarke, Katie Sidle, Rickie Patani

**Affiliations:** 1 Department of Neuromuscular Disease, UCL Queen Square Institute of Neurology, Queen Square, London WC1N 3BG, UK; 2 The Francis Crick Institute, 1 Midland Road, London NW1 1AT, UK

**Keywords:** astrocytes, prion-like, Alzheimer’s disease, Parkinson’s disease, amyotrophic lateral sclerosis

## Abstract

Accumulating evidence suggests that neurodegenerative diseases are not merely neuronal in nature but comprise multicellular involvement, with astrocytes emerging as key players. The pathomechanisms of several neurodegenerative diseases involve the deposition of misfolded protein aggregates in neurons that have characteristic prion-like behaviours such as template-directed seeding, intercellular propagation, distinct conformational strains and protein-mediated toxicity. The role of astrocytes in dealing with these pathological prion-like protein aggregates and whether their responses either protect from or conspire with the disease process is currently unclear. Here we review the existing literature implicating astrocytes in multiple neurodegenerative proteinopathies with a focus on prion-like behaviour in this context.

## Teaser

Astrocytes are increasingly recognised as playing fundamental roles in a range of neurodegenerative diseases. Smethurst *et al*. review the role of astrocytes specifically in prion-like mechanisms of neurodegeneration, across a spectrum of neurological disorders.

## Introduction

The main component tying together many neurodegenerative diseases is the accumulation and deposition of misfolded proteins. The pathogenic factors underlying the cytotoxic role of aggregated and misfolded proteins are predicted by the prion hypothesis. There are four main behavioural characteristics that make a protein ‘prion-like’: (i) template-directed seeding and amplification of misfolded proteins to form aggregates; (ii) intercellular spreading of aggregates and propagation of the templated seeding reaction to nearby cells; (iii) conformational strains that produce distinct clinical and biochemical disease entities; and (iv) protein-mediated cellular toxicity.^1^ Indeed, on this last point there is mounting evidence to show that oligomeric intermediate species of these proteins may be responsible for the resultant neurotoxicity driving the disease.^2^^,^^3^ Importantly, although many neurodegenerative disease-related proteins possess ‘prion-like’ behavioural characteristics,^1^ they do not, however, typically possess the same high levels of infectivity and aggressive phenotype as seen with actual prion diseases, and are thus termed ‘prion-like’.

Currently, the differential response of neurons and astrocytes to aggregated proteins with prion-like behaviour remains relatively understudied. Specifically, there is conflicting evidence about the presence of aggregated proteins in astrocytes at post-mortem, how these aggregates affect astrocytes and what role they play in neurodegeneration.^4-9^ Importantly, previous landmark studies have characterized the neurotoxic and neuroprotective roles of astrocytes.^10–12^ These have been followed up by innumerable reports further demonstrating these findings and their possible mechanistic basis across different neurodegenerative diseases.^13–18^ Recent work also suggests that astrocyte reactive transformation can occur cell autonomously in the context of neurodegeneration-causing gene mutations.^19^ The purpose of this article is to review the literature that directly explores the role of astrocytes in proteinopathies with prion-like behaviour in the following neurodegenerative diseases: prion disease, Alzheimer’s disease, tauopathies, synucleinopathies, Huntington’s disease and amyotrophic lateral sclerosis (ALS).

## Prion disease

Prion diseases, also known as transmissible spongiform encephalopathies, are a group of fatal neurodegenerative diseases characterized by the deposition of misfolded and aggregated prion protein (PrPSc) in the CNS, often causing severe cerebellar ataxia and rapidly progressive dementia. This is pathologically characterized at post-mortem by spongiosis (focal or diffuse clusters of small vacuoles in the neuropil of the deep cortical layers, subcortical grey matter or cerebellar cortex), reactive transformation of astrocytes, microglial activation and neuronal death. These pathologies result in a clinically heterogeneous set of conditions, whereby certain strains of the protein result in different misfolded conformations of aggregated PrPSc in distinct CNS regions.^20^ These different strains of misfolded prion protein result in distinct diseases characterized by unique biochemical and clinical features that include: Creutzfeldt–Jakob disease, kuru, fatal familial insomnia and Gerstmann–Sträussler–Scheinker syndrome. In addition to these human diseases, prions also cause disease in other species including cattle (bovine spongiform encephalopathy), deer (chronic wasting disease), sheep and goats (scrapie). There is now increasing evidence to suggest a dissociation between the aggregation of PrPSc and toxicity. This disconnect may be explained by the formation of intermediate toxic oligomeric species that have been described both *in vitro* and *in vivo* for the prion protein.^21^ Indeed, aggregates are unlikely to be the active pathological species, but rather a marker that a cell has been exposed to a toxic, misfolded protein. These pathological protein states may consist of a pool of fibrils or oligomers and are most appropriately termed ‘assemblies’ to distinguish these toxic entities from the inert end state aggregates.^1^ Importantly, it has also been suggested that PrP toxicity can be triggered by the monomeric form, independently of its oligomeric state.^22^ Furthermore, the N-terminus of PrP has been shown to function as a toxic effector mediating disease-related spontaneous ionic currents, regulated by the C-terminus.^23^

PrPSc aggregates were first detected in astrocytes in scrapie infected sheep and were noted to form prior to neuronal aggregates and before the development of the cardinal scrapie pathological features (reactive transformation of astrocytes, vacuolation, neuronal loss and amyloid deposition).^24^ This alluded to the potential role of astrocytes in attempting to alleviate pathology or in promoting the replication and spread of the pathological protein. A later study in rodents identified high levels of PrPc expression in astrocytes and oligodendrocytes and provided further evidence to support the role of glia in PrPSc propagation.^25^ In mice expressing a hamster PrP under an astrocyte-specific promoter, infection with hamster scrapie led to high levels of PrPSc infection with the mice exhibiting a severe disease phenotype.^26^ Indeed, detailed microscopy studies of prion pathology in the cerebellum of scrapie infected sheep have also highlighted the ability of astrocytes to sustain active prion infection with glial PrPSc pathology being detected in all cases.^27^^,^^28^ A further technical advance using flow cytometry allowed the identification of PrPSc in neurons, astrocytes and microglia early on during prion infection in mice.^29^ Collectively, these findings highlight a clear involvement of astrocytes in the propagation of infectivity and induction of prion disease. Another study using hamster scrapie as a model showed that the 263K strain induced PrPSc in ∼90% of reactive astrocytes suggesting that astrocytes may be able to produce PrPSc themselves, initiating a mechanism that may further trigger reactive transformation of astrocytes and drive the spread of both pathology and toxicity.^30^ Interestingly, however, a follow-up study from the same group using the same model also reported PrPSc aggregation in neurons (in the absence of PrP expression) and in the extracellular neuropil space. While they detected reactive changes in the astrocytes, no further astrocytic phenotypes were reported.^4^ Further work has subsequently demonstrated cell type specific differential toxicity when comparing neuron and astrocyte-specific expression of PrP and scrapie infection. Notably, animals with neuron specific expression succumbed to disease in 100 days compared to 360 days for astrocyte-specific expression.^5^ These data suggest that while neurons remain the predominant pathologically affected cell types in prion disease, astrocytes are also involved in driving this pathology, at least to some degree.

Astrocyte PrPSc infectivity has also been replicated in rodent *in vitro* models that induce neuronal death,^31^ and PrPSc pathology is actively taken up in human astrocyte cell lines from CJD patients where degradation of both proteinase K (PK) sensitive (PrPsen) and PK resistant (PrPres) forms of PrPSc was noted, highlighting a putative protective role for astrocytes in clearing prion disease pathology.^32^ Indeed, these data were corroborated from studies of hamster PrPres protein, which demonstrated that astrocytes and meningeal fibroblasts uptake PrPres more efficiently than neurons, potentially as a protective mechanism for protease digestion and clearance of infection.^33^ As a potential secondary effect of this, astrocytes could be propagating the resistant prion proteins to other cell types and aiding further infection. A more recent study showed that, in primary cultures of cerebellar neurons and astrocytes, the astrocytes were more prone to prion replication and accumulation of aggregated PrPSc and that prion pathology can be transferred between astrocytes and neurons through cell–cell contact occurring via tunnelling nanotubes.^34^ This tunnelling nanotube mechanism of spread and propagation may serve as both neuroprotective on one hand and also aid in sustaining prion infection on the other.

**Figure 1:**
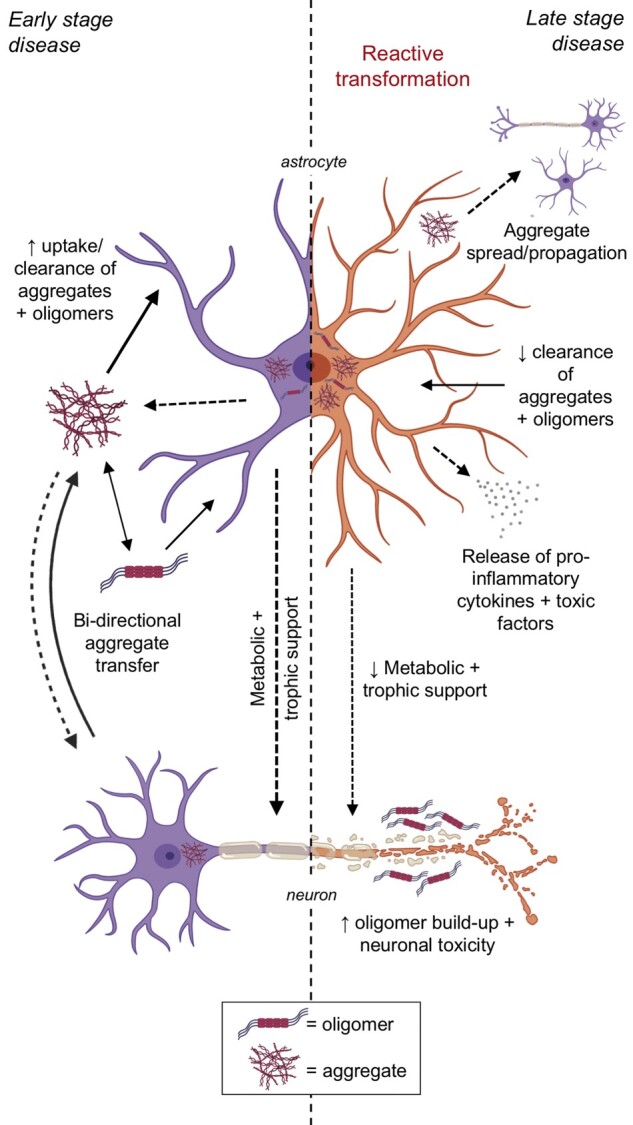
**Diagrammatic representation of hypothetical astrocytic involvement in prion-like mechanisms of early- and late-stage neurodegenerative disease.** The early stage represents an increased spread, uptake and clearance of aggregates and oligomers from neurons for degradation with continued metabolic and trophic support. Late-stage disease represents a decrease in aggregate and oligomer uptake, degradation and clearance, increased reactive astrocyte transformation, increased aggregate spread and propagation to other neurons and astrocytes and increased oligomer build up resulting in increased neuronal toxicity. Created with BioRender.com.

In the human prion diseases, the presence of a polymorphic variant (methionine/methionine, methionine/valine or valine/valine) at codon 129 of the prion gene (*PRNP*) can determine disease susceptibility and disease phenotype in patients with prion disease^35^ through the production of distinct conformers of misfolded PrP (‘strains’). One study demonstrated that multiple CJD prion strains can be replicated in human induced pluripotent stem cell (iPSC)-derived astrocytes in a PRNP codon 129 genotype dependent manner,^36^ providing further support to the role of astrocytes in potentially influencing disease susceptibility or phenotype. Together, these findings demonstrate that not only do astrocytes accumulate PrPSc in prion diseases, but they may also be driving early neuroprotective responses by clearing PrPSc infection. These neuroprotective responses may at the same time serve as a double-edged sword that can assist in sustaining the prion infection for long enough until the astrocytes become neurotoxic and exacerbate the pathological process by propagating infection at the later stages of disease (Fig. 1).

## Alzheimer’s disease: amyloid-β

Alzheimer’s disease is a progressive neurodegenerative disease, causing irreversible loss of neurons leading to a progressive cognitive decline. One of the hallmarks of Alzheimer’s disease is the deposition of aggregated misfolded amyloid-β (Aβ) protein as extracellular plaques in the neuropil. Amyloid-β is processed via amyloid precursor protein (APP) at the cell membrane where it is cleaved by gamma secretase into various fragments including Aβ40 and Aβ42. It is now well established that aggregated amyloid-β has prion-like behaviour due to its capacity for seeding, ability to spread and to propagate, form distinct strains and exert toxicity via its aggregated oligomeric intermediates both *in vitro* and *in vivo* (reviewed in Watts and Prusiner^37^). However, an important distinction in the context of amyloid-β, when compared to other proteins described in this review, is that it is accumulating extracellularly. Indeed, the most toxic species consists of soluble oligomeric assemblies, which are generated intracellularly and then secreted extracellularly, propagating via cell to cell spread.^38–41^

As amyloid-β plaques are extracellular in the human Alzheimer’s disease brain, they do not form in astrocytes. However, astrocytes have well known roles in the secretion, uptake and clearance of aggregated neuronal amyloid-β peptides.^41–44^ Furthermore, the soluble oligomeric aggregated species of amyloid-β, rather than insoluble fibrillar amyloid-β, are thought to be responsible for triggering neurotoxicity.^40^^,^^46–50^ While the impact of these oligomers is well understood in neurons, their effect on astrocytes is still not well characterized.

Reactive transformation of astrocytes is one of the earliest detectable features in Alzheimer’s disease. Astrocyte reactivity is accompanied by the release of pro-inflammatory cytokines such as TNF-α, IL-6 and IL1β, which are thought to mediate neurotoxic effects.^11^^,^^51^ Evidence suggests that amyloid-β oligomers can trigger the release of these cytokines more acutely than fibrillar forms, producing higher levels of inducible nitric oxide synthase (iNOS), nitric oxide (NO) and TNF-α in primary astrocyte cultures.^52^ Apolipoprotein E4 (ApoE4) is the most prevalent genetic risk factor for Alzheimer’s disease and is expressed in more than half of patients. Studies suggest that low levels of total apoE, as exhibited by ε4 carriers, may directly contribute to the disease progression.^53^ Furthermore, it has also been shown that toxicity of Aβ42 oligomers is exacerbated when neurons are co-cultured with ApoE4 knockout astrocytes.^54^ Interestingly, another study demonstrated that an injection of Aβ42 oligomers caused neuronal toxicity via mechanisms initiated by astrogliosis and reactive transformation rather than direct effects on neurons.^55^ Furthermore, Aβ42 oligomers were shown to induce astrocyte reactivity and also reduce astrocyte viability in primary cultures in a dose dependent manner.^55^^,^^56^ A more recent study from the same group identified specific nonamer and dodecamer soluble amyloid-β oligomers in the brains of mutant PSEN1 mice that exhibit cognitive decline. In this case, these oligomers were found to specifically amplify in astrocytes, where they induced and augmented neuronal damage non-cell autonomously through reactive astrocyte transformation.^57^

Mechanistically, this increase in astrocyte reactivity may be driven by Aβ42 oligomer disruption of ER calcium homeostasis leading to ER stress and reactive astrocyte transformation.^58^ Indeed, oligomeric Aβ40 and Aβ42 were found to specifically increase calcium signalling in astrocytes and not neurons. These oligomers were also found to increase levels of reactive oxide species (ROS), induce mitochondrial depolarization, reduce levels of the antioxidant GSH and cause metabolic dysfunction in astrocytes.^59^^,^^60^ This increase in ROS secretion from oligomer treated astrocyte cultures was also found to increase neuronal calcium signalling that in turn reduced protective STAT3 signalling.^61^ Some of this toxicity may be driven via mGluR5 receptor dependent ATP release from astrocytes,^61^^,^^62^ and activated scavenger receptors may be responsible for the phagocytic uptake of amyloid-β oligomers that drives glial toxicity via the hexapeptide XD4.^63^

Amyloid-β oligomers have been found to induce reactivity and reduce neuroprotective capacity in primary astrocyte cultures and *in vivo* via alteration in the secretion of the synaptogenic factor TGF-β1.^6^ Further neuroprotective benefits of astrocytes have been shown to be mediated by IGF1, which can release neuron bound amyloid-β oligomers and astrocyte oligomer treatment can reduce IGF1 signalling to neurons.^64^ In contrast, another study demonstrated that oligomeric amyloid-β was specifically toxic to neurons and not astrocytes even at the highest concentrations and that this toxicity was JUN-kinase dependent.^7^

The neuroprotective capacity of astrocytes against amyloid-β pathology may lie in some partial uptake of the aggregates for clearance during the early phases of amyloid-β aggregation, but once levels of aggregation are high enough, a neurotoxic role seems to supersede this protection and they become instrumental in the degenerative process. However, the role of astrocytes in the processing, seeding and transmission of amyloid-β pathology remains incompletely resolved and requires further investigation.

## Tauopathies

The term tauopathy refers to a collection of progressive age-related neurodegenerative diseases that are clinically and biochemically heterogeneous, but commonly involve the deposition of misfolded, aggregated and phosphorylated tau inclusions in the cells of the CNS. The tau protein aggregates in the form of neurofibrillary tangles (NFTs), pre-tangle deposits and neuropil threads within neuronal and astrocytic processes. The primary tauopathies include frontotemporal lobar degeneration (FTLD) with tau, corticobasal degeneration (CBD), Pick’s disease (PiD) and progressive supranuclear palsy (PSP), each of which can express various isoform ratios (3R and 4R), conformations and subcellular localization of tau pathology. While tau is predominantly expressed in neurons^65^^,^^66^ it is expressed at lower levels in glia (oligodendrocytes > astrocytes).^67^ Multiple lines of evidence now demonstrate that tau exhibits prion-like behaviour,^68^ and that astrocytes can actively participate in the spread and/or clearance of aggregated tau species.^65^

Alzheimer’s disease-related tau pathology may be distinct from other tauopathies given that tau aggregation in this context is considered a secondary event to the deposition of extracellular amyloid-β plaques. Furthermore, wild-type tau aggregation is present in Alzheimer’s disease-related tau pathology rather than tau aggregation driven by *MAPT* gene mutations that can cause other tauopathies.^69^ Tau aggregates in Alzheimer’s disease contain both 3R and 4R forms of tau deposited as intraneuronal neurofibrillary tangles, but astrocytic tau pathology is rarely detected.^69^ However, astrocyte reactivity is a well observed phenomenon in Alzheimer’s disease^70^ and is therefore thought to play a central role in disease pathogenesis. Studies examining the effect of any aggregated species, including oligomers and fibrils, from Alzheimer’s disease patients on astrocytes have yet to be conducted but will provide important insights.

Astrocytes exhibit a range of morphological phenotypes across different tauopathies including tufted astrocytes in PSP, astrocytic plaques in CBD, thorn shaped astrocytes, granular fuzzy astrocytes in ageing-related tau astrogliopathy (ARTAG), ramified astrocytes in PiD and astrocytes with globular inclusions in globular glial tauopathy. One of the first studies to note paired helical filament or phospho-tau S396/S404 positive tau pathology in aged mice demonstrated that astrocytes may be involved in the uptake and spread of tau pathology.^71^ Interestingly, oligodendrocytes are also known to form tau inclusions in the form of coiled bodies and occasionally globular inclusions.^72^ One study demonstrated that tau strains from CBD and PSP post-mortem material could seed specifically in oligodendrocytes and astrocytes. Additionally, glial tau seeding was achieved in non-transgenic mice, thereby implicating glial tau pathology in the progression of tauopathies.^73^ The same group reported that astrocytic tau pathology from CBD patients and tufted astrocyte pathology from PSP patients could be transmitted via their unique tau strains. Oligodendroglial tau seeding and spreading could propagate along white matter tracts in a neuronal tau knockdown (KD) mouse model whereas astrocytic tau pathology could not. These findings suggest that oligodendroglial tau pathology potentially has a distinct pathogenic impact when compared to astrocytic pathology.^74^ Furthermore, astroglial tau pathology from patients with ARTAG can be seeded to wild-type mice in neuronal and glial populations in the brain,^72^ and CBD astrocytic plaques and tufted astrocytes from PSP patients can also be transmitted via unique strains maintaining a glial cell type specificity for propagation.^75^ Together these data show that some tau ‘strains’ are more likely to affect glia than others and further implicate a nuanced glial involvement in tauopathies.

The effects of tau inclusions on the health of astrocytes are currently unknown and represent an interesting research avenue for future studies. As glial cells typically do not secrete or express much tau protein, the deposition of tau pathology in glial cells is an unusual phenomenon that may be explained by the uptake of neuron-derived monomeric and aggregated tau from the extracellular space. Uptake of extracellular tau by astrocytes cells can occur via many mechanisms^76^ with the uptake of fibrillar and aggregated species occurring mainly via the lysosomal pathway^77^^,^^78^ and heparan sulphate proteoglycans (HSPGs),^77–79^ and monomeric tau uptake via an unknown HSPG independent mechanism.^80^ Indeed, a recent paper identified low density lipoprotein receptor-related protein 1 (LRP1) as a novel receptor that mediates tau uptake *in vitro* and *in vivo* models of tau spread with a significant role in astrocytic (as well as neuronal) tau uptake.^79^ In the early stages of disease, this uptake may represent a protective mechanism becoming pathological at a later stage when cellular clearance mechanisms may become overwhelmed. Furthermore, recent data suggest that filamentous tau aggregates can activate integrin signalling in astrocytes where seeds can be taken up and induce further astrocytic activation to release a variety of pro-inflammatory chemokines and cytokines constituting a deleterious reactive astrocyte phenotype.^81^ Recent data showed that astrocytes were found to rapidly accumulate toxic tau oligomers in an APP-dependent manner whereby intracellular calcium signalling and calcium-dependent release of gliotransmitters were disrupted. This subsequently induced synaptic dysfunction in neighbouring neurons suggesting that astrocytes play a major role in Alzheimer’s disease pathology by exacerbating synaptic toxicity via the reduction of gliotransmitters.^82^ It should also be noted that astrocytic tau inclusions have been found in aged mice independent of dementia suggesting that astrocytes may also have a role in internalizing and clearing tau.^83–85^ However, further studies of astrocyte activity during the early and late stages of aggregation in *in vitro* co-culture models and *in vivo* models will shed more light on the temporal role of astrocytes in the pathogenesis in the tauopathies.

## Synucleinopathies

Parkinson’s disease, dementia with Lewy bodies (DLB) and multiple system atrophy (MSA) constitute a group of neurodegenerative conditions known as synucleinopathies, pathologically represented by the presence of aggregated α-synuclein protein. α-Synuclein is the main component of a pathological hallmark of synucleinopathies known as Lewy bodies and Lewy-like inclusions, found in the neurons of patients with Parkinson’s disease and DLB, and in the astrocytes and oligodendrocytes of patients with MSA.^86–88^ Indeed, α-synuclein inclusions in oligodendrocytes termed glial cytoplasmic inclusions (GCIs) are considered the pathological hallmark of MSA specifically, raising the hypothesis that glial cytoplasmic inclusions drive pathogenesis in this condition. Astrocytic α-synuclein pathology is less common than oligodendrocyte GCIs^89^ and is prevalent at later stages of Parkinson’s disease and DLB.^90^ Indeed, astrocytic α-synuclein is also found in 40% of MSA patients, predominantly in subpial and periventricular regions.^91^ These cell types have low levels of expression of α-synuclein and the mechanistic basis for the development of these inclusions is not completely understood.

α-Synuclein is a predominantly synaptic protein that is secreted from neuronal cells by exocytosis.^92^ Oligomeric forms of the protein were found to be elevated in CSF and blood plasma of Parkinson’s disease patients.^93^ One of the first papers to report the effects of α-synuclein on astrocytes showed that aggregated α-synuclein can be exocytosed from neurons and taken up by astrocytes, forming inclusions and causing an upregulation of inflammatory response genes.^94^ Subsequent studies demonstrated that various recombinant forms of α-synuclein could induce microglial and astrocyte reactivity, but toll-like receptor 4 (TLR4) was not required for uptake in astrocytes, unlike microglia.^95^^,^^96^ Another study demonstrated the uptake of recombinant α-synuclein into cortical neurons, astrocytes and fibroblasts, and reported mitochondrial dysfunction in these cell types.^97^ Using purified α-synuclein aggregates from human Parkinson’s disease brain tissue, high content imaging and microfluidics on primary rat cortical neurons and astrocytes, one group has shown that astrocytes can more readily uptake α-synuclein aggregates when compared to neurons via endocytosis. It was also seen that these aggregates could readily transfer between these cell types, resulting in neuronal death if transferred from astrocytes to neurons,^98^ thereby demonstrating a gain of toxic astrocyte function. Loria *et al*.^99^ interestingly demonstrated that this spread and propagation of α-synuclein fibrils was cell type dependent. They demonstrate that transfer of fibrillar alpha synuclein is most optimal from astrocyte to astrocyte and neuron to astrocyte but less efficient from astrocyte to neuron. Furthermore, astrocytes were able to more efficiently degrade this fibrillar alpha synuclein than neurons providing further evidence to support the hypothesis that astrocytes can capture and degrade pathological α-synuclein as a neuroprotective response.^99^ Another study demonstrated the accumulation of α-synuclein oligomers in human embryonic stem cell derived astrocytes, where failure of the lysosomal digestion pathway to process excess oligomers resulted in inclusion formation in the trans Golgi network, resulting in ER swelling and mitochondrial abnormalities. This study also demonstrated that upon oligomer accumulation, astrocytes formed tunnelling nanotubes to secrete accumulated oligomers to neighbouring healthy astrocytes. These data suggest a potential early protective mechanism and role for astrocytes in the transfer of α-synuclein in the disease process.^8^

Another study introducing monomeric, oligomeric and fibrillar forms of synuclein to primary rat astrocyte cultures described changes akin to reactive transformation such as morphological changes, and an increase in intracellular ROS, cytokine release and GFAP expression.^9^ Here, co-culture experiments revealed significant astrocyte-derived cytotoxicity to neurons, with fibrillar α-synuclein-treated astrocytes inducing the highest levels of toxicity. However, oligomeric α-synuclein treatment was the only condition capable of inducing mitochondrial dysfunction and increased hydrogen peroxide production within astrocytes. The levels of TNF-α and cytokine gene expression also varied depending on the species of α-synuclein used. Cumulatively, this study suggested that both oligomeric and fibrillar species potentially contribute to astrocyte mediated toxicity, but through distinct mechanisms and potencies.^9^ Interestingly, different morphological subtypes of α-synuclein inclusion (filamentous and granular) have also been demonstrated within primary astrocyte cultures derived from mutant (A53T or A30P) human α-synuclein transgenic mice exposed to MSA brain homogenate. This study suggested that α-synuclein expression levels determine selective tropism of aggregation.^100^

Collectively, these data present a mixed picture of astrocytes playing both a protective and a deleterious role in response to α-synuclein aggregation. Indeed, many of these observations can potentially be attributed to differences in the models used for each study including species (both of cell model and inoculum), astrocyte subtype and duration of treatment. The key to elucidating the role of astrocytes in α-synuclein aggregation induced toxicity lies in further investigation using more physiologically relevant cell types such as human iPSC-derived region-specific astrocytes in co-culture with neurons.^101–103^

## Huntington’s disease

Huntington’s disease is an autosomal dominant neurodegenerative disease caused by a CAG expansion within the huntingtin gene (*HTT*) that leads to the development of a movement disorder, psychiatric and cognitive symptoms. Translated polyglutamine expansions in the huntingtin protein, if higher than 35 repeats, lead to the aggregation of huntingtin (HTT) protein and the development of Huntington’s disease. A pathological hallmark of Huntington’s disease is the presence of aggregated HTT in the form of neuronal intranuclear inclusions located in the striatum. It is thought that such pathology causes the substantial degeneration of medium spiny neurons, but HTT aggregation has also been identified in astrocytes, and its role in these cells as part of the disease process is still not clear.^97^^,^^98^^,^^104–109^ Increasing evidence now suggests that HTT also demonstrates prion-like behaviour including the spreading and propagation of HTT aggregates in multiple *in vitro* cellular models (reviewed in Bradford *et al*.^106^). However, the role of astrocytes in response to this prion-like behaviour of HTT is still not well understood and may provide key insight into the pathogenesis and therapeutic opportunities for Huntington’s disease.

Early evidence suggested that glial expression of mutant HTT containing polyQ expansion of 115–150 repeats induces its accumulation in glial nuclei in Huntington’s disease brains, accompanied by a decrease in glutamate transporters and glutamate uptake in glial cultures and mouse Huntington’s disease brains.^105^ Wild-type glia were found to be neuroprotective in co-culture models, whereas neurons cultured with mutant Huntington’s disease glia were vulnerable to glutamate-induced excitotoxicity.^110^ The same group developed a transgenic Huntington’s disease model with astrocyte-specific expression of the more toxic N-terminal HTT fragments (160Q) which displayed an age-related motor phenotype and glutamate transporter deficiency.^110^^,^^111^ This mouse model was crossed with another neuronally expressed polyQ HTT repeat mouse, resulting in a more severe phenotype and increased vulnerability to excitotoxic stress.^112^ One group used adeno-associated virus expression of the N-terminal (1–552) fragment of HTT with 100Q repeats in primary cortical neurons and found that some astrocytes developed HTT inclusions that sequestered clathrin and Golgi complexes, resulting in a significant decrease in secretion of mature brain-derived neurotrophic factor (BDNF), which in turn disrupted neurite development in primary cortical neurons.^113^ A more recent study using primate iPSC-derived astrocytes from transgenic mutant HTT (mHTT) monkeys also demonstrated the formation of glial HTT inclusions which resulted in a reduction in the following parameters: expression of superoxide dismutase 2 (SOD2) (a mitochondrial enzyme that converts superoxide radicals into hydrogen peroxide) and peroxisome proliferator activated receptor γ (PPARγ) co-activator 1α (PGC1) (a regulator of mitochondrial biogenesis and oxidative stress), glutamate uptake, 4-aminopyridine (4-AP) response (a non-selective voltage dependent K^+^ channel blocker), and aberrant electrophysiological responses.^114^ Taken together, these data demonstrate that mHtt aggregates can readily form in astrocytic populations and probably influence pathogenesis via alteration of mitochondrial responses to oxidative stress and neuronal activity.

The prion-like transmission of HTT aggregates from neurons to glia was first demonstrated in a *Drosophila* model of Huntington’s disease.^115^ Here, glia were shown to regulate the number of HTT aggregates in neurons through a clearance mechanism mediated via Draper signalling. Draper is the *Drosophila* orthologue of *MEGF10* in mammals, and is an engulfment receptor that regulates the clearance of cellular debris. These data indicate that glia may be contributing to the clearance and transmission of aggregates throughout the CNS and that modulating MEGF10 signalling may be a potential therapeutic target for reducing spread. Another study using a mouse model with basal ganglia restricted expression of N-terminal HTT fragments in either astrocytes, neurons or both demonstrated that astrocytes are less vulnerable to HTT aggregates. The study also showed that mutant HTT in neurons affected key components of the glial glutamate-glutamine cycle, at least at the mRNA level.^116^ Some of the mechanistic insights into this increased resistance of astrocytes to mutant HTT aggregates may arise from the increased rate of their degradation in astrocytes compared to neurons, increased levels of proteasome function in clearing soluble K48 linked ubiquitinated HTT,^117^ increased expression of heat shock proteins such as Hsp70 and C-terminus Hsp70 interacting protein (CHIP) and a reduced expression of HSPB1 protein (a CHIP inhibitory protein) compared to neurons.^117^^,^^118^ The evidence for the role of astrocytes and their functional handling of HTT is still in its infancy and requires further investigation; however, these aforementioned data suggest astrocytes may be contributing to disease progression and toxicity, and do appear to play a key role in spreading and propagation of HTT aggregates in the disease process.

## TDP-43 proteinopathies

Trans active DNA response binding protein (TARDBP) with a molecular weight of 43 kDa (TDP-43) is a DNA and RNA binding protein. Its abnormal cytoplasmic aggregation is considered the pathological hallmark of ∼97% of cases of ALS and ∼50% of cases of fronto-temporal lobar degeneration (FTLD-TDP), which have now been reclassified as TDP-43 proteinopathies. Mutations in the *TARDBP* gene are also causative of a small percentage of ALS and FTLD cases giving TDP-43 a central role in the pathogenesis of ALS and FTLD.^119–121^

TDP-43 is a predominantly nuclear protein that can dynamically shuttle between the nucleus and the cytoplasm. In ALS and FTLD it becomes mislocalized to the cytoplasm where it is cleaved, hyperphosphorylated and aggregated. The presence of aggregated TDP-43 is well documented in neurons and is also a common feature in oligodendrocytes.^122–124^ However, the presence of TDP-43 pathology in astrocytes is not well documented and less well studied in ALS and FTLD-TDP. Interestingly, astrocytic TDP-43 pathology is a key feature of Alexander disease, a condition caused by mutations in *GFAP*,^125^ but in this case TDP-43 is not cleaved into C-terminal fragments like in ALS and FTLD-TDP. TDP-43 pathology is also recognized in white matter astrocytes in FTLD-Tau, CBD,^126^ Cockayne syndrome^127^ and brain tumours^128^ suggesting that astroglial TDP-43 pathology does occur in some neurological diseases, but that its pathological relevance is unclear in ALS. *In vitro*, human iPSC-derived astrocytes with a TDP-43 M337V mutation have been reported to show cytoplasmic TDP-43 mislocalization and a cell autonomous survival phenotype.^129^ However, the generalizability of astrocytic cytoplasmic TDP-43 proteinopathy requires further systematic investigation across different model systems.

There is mounting evidence to demonstrate that TDP-43 is also a prion-like protein demonstrating many features of prion-like behaviour,^130^ yet few studies examining the pathological relevance astrocytes in this context have been undertaken. One group used rat neural stem cells differentiated to neurons, astrocytes and oligodendrocytes that virally overexpressed TDP-43 and used proteasome inhibition to induce TDP-43 aggregates in all three cell types. With time lapse imaging they could detect the formation of these aggregates in all three cell types, which was followed by cell death after 72 h and spreading of aggregates to neighbouring cells.^131^ This study indeed suggested that the aforementioned cell types are susceptible to pathological TDP-43 protein spread and related toxicity. To begin to address the differences in spread and susceptibility to toxicity between these cell types, we recently demonstrated that control human spinal iPSC-derived motor neurons and astrocytes can seed TDP-43 pathology from serially passaged ALS spinal cord extracts.^132^ We also showed that astrocytes were comparatively resistant to TDP-43 seeding, seeding-induced toxicity and recombinant TDP-43 oligomer-induced toxicity compared to motor neurons. Indeed, upon co-culture with motor neurons we demonstrated that these seeded aggregates can spread between both cell types with a predilection for motor neuron to astrocyte spread. The astrocytes were also neuroprotective to the motor neurons with no deleterious reactive astrocyte changes detected after co-culture.^132^ These data indicate that astrocytes are less vulnerable to TDP-43 protein-mediated toxicity and can—at least on initial exposure—be neuroprotective by clearing aggregated TDP-43 from motor neurons. However, at later disease stages they may be unable to clear aggregates and may participate in the spread and neurotoxic process. The elucidation of the exact mechanisms of these differential responses of neurons and astrocytes to TDP-43 pathology require further investigation and may highlight novel therapeutic targets to ameliorate protein-mediated toxicity.

## Conclusion

Multiple lines of evidence discussed herein implicate astrocytes as key players in proteinopathies in terms of their roles in developing/uptaking, transmitting and clearing pathological protein aggregates in multiple neurodegenerative diseases (Table 1). In addition, they may develop unique responses to protein aggregation where they can display a dual role in mounting initial neuroprotective responses followed by deleterious activity later in the disease process (Fig. 1). The delineation of these exact mechanisms and pathways are exciting areas of research and could lead to the discovery of much needed clinically impactful disease-modifying therapies for these devastating and incurable conditions.

**Table 1:** Astrocytes are increasingly recognized as playing fundamental roles in a range of neurodegenerative diseases

Common astrocyte-specific pathogenic mechanisms	Proteinopathy (disease-specific protein)					
	Prion disease (PrPsc)	Alzheimer’s disease (amyloid-β)	Tauopathies (Tau)	Synucleinopathies (α-synuclein)	Huntington’s disease (HTT)	Amyotrophic lateral sclerosis (TDP-43)
Reactive transformation	**✓**	**✓**	**✓**	**✓**	**✓**	**✓**
Increased protein uptake	**✓**	**✓**	**✓**	**✓**		
Accumulation of misfolded protein aggregates/disrupted clearance	**✓**	**✓**	**✓**	**✓**	**✓**	**✓**
Protein transfer/propagation	**✓**		**✓**	**✓**	**✓**	
Astrocyte-derived toxicity to neurons		**✓**	**✓**	**✓**		
Mitochondrial/metabolic dysfunction		**✓**		**✓**		
Disrupted Ca^2+^ homeostasis		**✓**	**✓**			

## Funding

This work was supported by the Francis Crick Institute which receives its core funding from Cancer Research UK 65 (FC010110), the UK Medical Research Council (FC010110) and the Wellcome Trust (FC010110). R.P. holds an MRC Senior Clinical Fellowship (MR/S006591/1) and a Lister Research Prize Fellowship.

## Competing interests

The authors report no competing interests.

## Abbreviations

ALS: amyotrophic lateral sclerosis
CBD: corticobasal degeneration
FTLD: frontotemporal lobar degeneration
PSP: progressive supranuclear palsy
